# Annotations

**Published:** 1906-07-21

**Authors:** 


### ANNOTATIONS.

### Hay Fever and its Cures.

As with sea-sickness, so with hay fever, the number of reputed cures is a good index of the caprice and inveteracy of the affection. There is hardly a disease known to medicine which manifests such caprice as does hay fever, but there are two constant factors in its manifestations—namely, the time of its appearance and the character of the symptoms. But in any given case the liability to recurrence and the response to treatment entirely pass the bounds of medical prophecy. Some people live a long life of immunity to fall victims in their dotage, while, conversely, not a few throw off without warning a susceptibility which has made life a burden, for the time being, over a long period of years. Similarly, as regards the influence of remedies an equal caprice is often found to obtain. A sufferer who has exhausted the catalogue of vaunted snuffs and sprays, and has very likely sacrificed large portions of his nasal mucous membrane to the cautery, comes suddenly upon a new-fangled compound which appears to act like a charm. His delight finds expression in free recommendations of the cure to his follow-sufferers, only perhaps to be exchanged for a gloom of corresponding intensity when he finds that in some fatal year the efficiency of the remedy is gone. The very latest thing in cures is "cold storage." It comes, as one might almost have guessed, from across the Atlantic. A gentleman who was a great victim to hay fever happened one summer to spend two hours in the refrigerating hold of a steamer. This cured him, and he had no further attacks that summer. Thenceforward, whenever an attack threatened he beat a hasty retreat to a cold-storage chamber and was cured. The idea is simple and pretty, but still in its infancy and awaiting judgment. It is quite likely to be as successful as other cures, with the single exception of a sea voyage. This we have never known to fail.

### Humanitarianism and War.

It would be interesting to have the opinion of some dispassionate observer from another planet, say one of Mr. Wells's Martians, upon the paradoxical attitude of civilised nations towards war. In a world of artificiality and carefully nurtured sentimentalism the natural man is kept so much in abeyance that the natural method of settling international difficulties, namely the appeal to brute force, seems an unspeakable anachronism. And so in fact it is. We have, centuries ago, discarded the settlement of individual differences by dint of the strong arm, yet in international matters we find ourselves forced to admit this most vulgar and illogical vindication of national claims. It is no wonder then that national acceptance of war as an inevitable evil should be tempered by individual bias against force, the civilised world over, and that in consequence an attempt should be made to mitigate the brutality of a proceeding whose brutality is its most conspicuous attribute. We are not here concerned to weigh the cruelties of war against the cruelties of peace, such, for instance, as the sweating of the defenceless worker, or the conscienceless oppression which has made commercial and financial morality a byword amongst the peoples. From this standpoint much might be said on behalf of war, but it is beside our text. The necessity for wars must be our postulate, and our strange duty is to kill or maim our enemies in the kindliest possible way. Side by side with this curious attitude of altruism go the attempts made to alleviate the wounded on the field, a humanitarianism of much less mingled motives. The latest suggestion to this end comes from Germany. Professor Schleich proposes that every soldier in the field should be provided with small aluminium capsules containing a narcotising mixture, sufficient to relieve pain during the interval between the infliction of the wound and the arrival of medical assistance. The idea seems at first sight to be inherently impracticable, but it affords an interesting reflection of the trend of civilisation in the matter of war.

### Multiple Telangiectases and Recurrent Epistaxis.

This condition, as the name implies, is characterised by the presence of more or less numerous spots or patches of dilated blood-vessels in association with attacks of nose bleeding. The telangiectatic areas are found on the skin, mostly of the face and fingers, and also on the buccal and nasal mucous membranes, and it is their presence in the last-mentioned which is presumably responsible for the epistaxis. The first suggestion which occurs in connection with these cases is that they are examples of the hæmorrhagic diathesis, and this receives apparent support from the fact that in the recorded instances several members of the same family have been affected. Opposed to this, however, is the distribution of the disease in both sexes (as compared with the preference of hæmophilia for the male sex) and the absence of any tendency to excessive bleeding on the occurrence of slight injuries. It would seem that the condition is a very rare one, and it is thus desirable to draw attention to it in the hope that further observations may be made and the true pathology determined. So far, some eight cases have been put on record, the last two being described and illustrated by Dr. A. Brown Kelly in the June number of the *Glasgow Medical Journal*. The patients were sisters, and it is to be noted that one of them died suddenly from syncope induced by very severe and persistent epistaxis. Dr. Kelly's paper is a very thorough and interesting discussion of the whole subject, and includes a study not only of his own cases, but also of those published by Professor William Osler, Dr. C. O. Hawthorne, and Rendu. It would seem desirable to have general attention called to a diseased condition which is of so obscure a nature, and to which serious practical contingencies are attached.

